# Discovery of novel biomarkers and phenotypes by semantic technologies

**DOI:** 10.1186/1471-2105-14-51

**Published:** 2013-02-13

**Authors:** Carlo A Trugenberger, Christoph Wälti, David Peregrim, Mark E Sharp, Svetlana Bureeva

**Affiliations:** 1 InfoCodex AG, Semantic Technologies, Bahnhofstrasse 50, Buchs (SG), CH-9470, Switzerland; 2 Merck Research Laboratories, 126 East Lincoln Avenue, Rahway, NJ 07065, USA; 3 Thomson Reuters, 5901 Priestly Drive, STE 200, Carlsbad, CA, 92008, USA

**Keywords:** *In silico* drug research, Semantic technologies, Text mining, Biomedical ontologies, Discovery of novel relationships

## Abstract

**Background:**

Biomarkers and target-specific phenotypes are important to targeted drug design and individualized medicine, thus constituting an important aspect of modern pharmaceutical research and development. More and more, the discovery of relevant biomarkers is aided by *in silico* techniques based on applying data mining and computational chemistry on large molecular databases. However, there is an even larger source of valuable information available that can potentially be tapped for such discoveries: repositories constituted by research documents.

**Results:**

This paper reports on a pilot experiment to discover potential novel biomarkers and phenotypes for diabetes and obesity by self-organized text mining of about 120,000 PubMed abstracts, public clinical trial summaries, and internal Merck research documents. These documents were directly analyzed by the InfoCodex semantic engine, without prior human manipulations such as parsing. Recall and precision against established, but different benchmarks lie in ranges up to 30% and 50% respectively. Retrieval of known entities missed by other traditional approaches could be demonstrated. Finally, the InfoCodex semantic engine was shown to discover new diabetes and obesity biomarkers and phenotypes. Amongst these were many interesting candidates with a high potential, although noticeable noise (uninteresting or obvious terms) was generated.

**Conclusions:**

The reported approach of employing autonomous self-organising semantic engines to aid biomarker discovery, supplemented by appropriate manual curation processes, shows promise and has potential to impact, conservatively, a faster alternative to vocabulary processes dependent on humans having to read and analyze all the texts. More optimistically, it could impact pharmaceutical research, for example to shorten time-to-market of novel drugs, or speed up early recognition of dead ends and adverse reactions.

## Background

### New frontiers for *in silico* drug research

Pharmaceutical research is undergoing a profound change. Over the last 10 years productivity has been steadily declining despite rising R&D budgets. Pipelines are drying up and there has been much talk of the end of the “blockbuster era” [1]. Recent trends by the largest companies in the pharmaceutical industry to outsource science are leading to contract research organizations (CRO) controlling significant processes and thusly, information.

Traditionally, drugs are discovered in natural products by happenstance or, more recently, by synthesizing and screening large libraries of small molecule compounds (combinatorial chemistry). Both cases involve time-consuming multi-step processes to identify potential candidates according to their pharmacokinetic properties, metabolism and potential toxicity. The advent of more computational approaches such as genomics, proteomics and structure-based design has revolutionized this process. Today, computational methods permeate many aspects of drug discovery. High-performance computers and data management and analysis software are being applied to the transformation of complex biomedical data into workable knowledge driving the drug discovery process [1,2].

On this stage, data come in two types: structured, identifiable data organized in a well-defined structure (typically a database, table or hierarchical scheme) and unstructured, with no identifiable organization. Typically, numerical values from sensors and other types of measurements constitute an example of structured data, while free text falls in the unstructured data category. While the major data mining effort, in both scientific and business applications (such as genomics/proteomics and customer behavior/churning, respectively) has focused on structured data, it has been estimated [3] that 85% of the data stored on the world’s computers are unstructured. However, the main (and best known) automated manipulation of unstructured data today is restricted to “search” (information retrieval; IR), in both its classical form based on keywords or in its more advanced versions relying on machine intelligence and statistics. The extraction of information by semantic analysis of content is still left to the ingenuity of the human reader.

The pharmaceutical industry is no different. The bulk of the computational effort goes into crunching molecular data that becomes available through advances in crystallography, nuclear magnetic resonance (NMR) and bioinformatics. Techniques like virtual screening, *in silico* absorption/distribution/metabolism/excretion (ADME) prediction and structure-based drug design are all aimed at leading discovery by identifying suitable interactions in large molecular databases [4],

Biochemical structures are not the only data being amassed. The sheer numbers of research publications accumulating in public as well as proprietary repositories are such that no human team, however specialized, can easily maintain an up-to-date overview. PubMed, one of the most important repositories, alone has reached the level of 19 million documents, growing at the rate of over one per minute. Semantic technologies attempt to make these large collections of unstructured data more tractable, with text mining representing the most important class. The main thrust in health care text mining concerns “information extraction” (IE), whose goal consists in identifying mentions of named entity types and their explicitly lexicalized, semantically typed relations. This is the typical domain of natural language processing (NLP) systems and there is already a sizable body of literature on this subject (for a review see [5,6]). A harder task is what has also been dubbed [5] “the holy grail of text mining knowledge discovery” (KD) where the aim is to find new pieces of information which, unlike in the IE/NLP scenario, are not already explicitly stated in available documents and have to be discovered by associative, semantically unspecified relationships. Knowledge discovery is the main subject of the present paper.

There are a few systems addressing this grand challenge [5,6]; however, a canonical methodology has not emerged. Merck & Co., Inc., has for many years explored advanced search of unstructured information for purposes of drug discovery and development. This paper reports on a knowledge discovery text mining pilot project employing the autonomous, self-organized semantic engine InfoCodex. The high-level goal of the project was to explore the power of semantic machine intelligence for the screening of a collection of research documents in search of unknown/novel information relevant to early-stage drug candidate discovery and development. The specific task was to discover unknown/novel biomarkers and phenotypes for diabetes and/or obesity (D&O) by semantic machine analysis of diverse and numerous biomedical research texts.

### Focus on biomarkers and phenotypes

In order to stem declining revenues the pharmaceutical industry is restructuring and exploring new business models. Drugs of the future will be targeted to populations and groups of individuals with common biological characteristics predictive of drug efficacy and/or toxicity. This practice is called “individualized medicine” or “personalized medicine” [1,6]. The characteristics are called “biomarkers” and/or “phenotypes”.

A biomarker is a characteristic that is objectively measured and evaluated as an indicator of normal biologic processes, pathogenic processes, or pharmacologic responses to a therapeutic intervention. In other words, a biomarker is any biological or biochemical entity or signal that is predictive, prognostic, or indicative of another entity, in this case, diabetes and/or obesity.

A phenotype is an anatomical, physiological and behavioural characteristic observed as an identifiable structure or functional attribute of an organism. Phenotypes are important because phenotype-specific proteins are relevant targets in basic pharmaceutical research.

Relevant examples of biomarkers/phenotypes and their vital discovery outcomes are: HER2 for breast cancer, BCR-ABL kinase and tyrosine-protein kinase Kit for chronic myloid leukemia, and abnormal or mutated BRCA1 or BRCA2 gene for breast, pancreatic, testicular, or prostate cancer.

Biomarkers and phenotypes take on an increasingly important role for identifying target populations stratified into subgroups in which the efficacy of specific drugs is maximized. For individuals outside this target, the drug might work less efficiently or even cause undesired side effects. Avastin is an often cited example of some patients responding well to a drug while others experience adverse effects, where careful biomarker research might have led to an entirely different regulatory outcome [1].

Biomarkers and phenotypes constitute one of the “hot threads” of diagnostic and drug development in pharmaceutical and biomedical research, with applications in early disease identification, identification of potential drug targets, prediction of the response of patients to medications, help in accelerating clinical trials and personalized medicine. The biomarker market generated $13.6 billion in 2011 and is expected to grow to $25 billion by 2016 [7].

At odds with this trend are recent reports that biomarkers “are either completely worthless or there are only very small effects” in predicting, for example, heart disease [8]. Ongoing and future efforts to validate or disprove these conclusions within the scientific community magnify the importance of examining the immense volumes of biomarker research and observational study data.

## Methods

### High-level description of the experiment

The object of the experiment was for the InfoCodex semantic engine to discover unknown/novel biomarkers and phenotypes for diabetes and/or obesity (D&O) by analysis of a diverse and sizable corpus of unstructured, free text biomedical research documents. The engine and the corpus are described in greater detail below. Briefly, the corpus consisted of approximately 120,000 PubMed [9] abstracts, ClinicalTrials.gov [10] summaries, and Merck internal research documents. The D&O related biomarkers and phenotypes were then compared with Merck internal and external vocabularies/databases including UMLS [11], GenBank [12], Gene Ontology [13], OMIM [14], and the Thomson Reuters [15] D&O biomarker databases according to precision, recall, and novelty.

### The InfoCodex semantic engine

InfoCodex is a text analysis technology designed for the unsupervised semantic clustering and matching of multi-lingual documents [16]. It is based on a combination of a universal knowledge repository (the InfoCodex Linguistic Database, ILD), statistical analysis and information theory [17], and self-organizing maps (SOM) [18].

#### InfoCodex linguistic database [ILD]

The ILD contains multi-lingual entries (words/phrases), each characterized by:

• its type (noun, verb, adjective, adverb/pronoun, name)

• its language (en, de, fr, it, es)

• its significance rank from 0 (meaningless glue word) to 4 (very significant and unique)

• a hash code for the accelerated recognition of collocated expressions.

The words/phrases with almost the same meaning are collected into cross-lingual synonym groups (microscopic semantic clouds) and systematically linked to a hypernym (taxon) in a universal 7-level taxonomy (simplified ontology restricted to hierarchical relations).

With its 3.5 million classified entries, the ILD corresponds to a very large multi-lingual thesaurus (for comparison, the *Historical Thesaurus of the English Oxford Dictionary*, often considered the largest in the world, has 920,000 entries). The content and the semantic structure of the ILD are largely based on WordNet [19], combined with some 100 other well established knowledge sources.

#### Text mining and content analysis

The words/phrases found in a document are matched with the entries in ILD, providing a cross-language content recognition. The taxons most often matched by a document represent the document’s main topics. Using statistical methods and information theoretical principles, such as entropies of individual words, a 100-dimensional content space is constructed that can depict the document characteristics in an optimal way. The documents are then projected into this content space, resulting in 100-dimensional vectors characterizing the individual documents together with a generated set of the most relevant synonym groups.

#### Categorization of a document collection (Kohonen Map)

The fully automatic categorization is achieved by applying the neural network technique of Kohonen [18], which creates a thematic landscape according to and optimized for the thematic volume of the entire document collection. Prior to starting the unsupervised learning procedure, a coarse group rebalancing technique is used to construct a reliable initial guess for the SOM. This is a generalization of coarse mesh rebalancing [20] to general iterative procedures, with no reference to spatial equation as in the original application to neutron diffusion and general transport theory in finite element analysis. This procedure considerably accelerates the iteration process and minimizes the risk of getting stuck in a sub-optimal configuration.

For the comparison of the content of different documents with each other and with queries, a similarity measure is used which is composed of the scalar product of the document vectors in the 100-dimensional content space, the reciprocal Kullback–Leibler distance [21] from the main topics, and the weighted score-sum of common synonyms, common hypernyms and common nodes on higher taxonomy levels.

**Figure 1:**
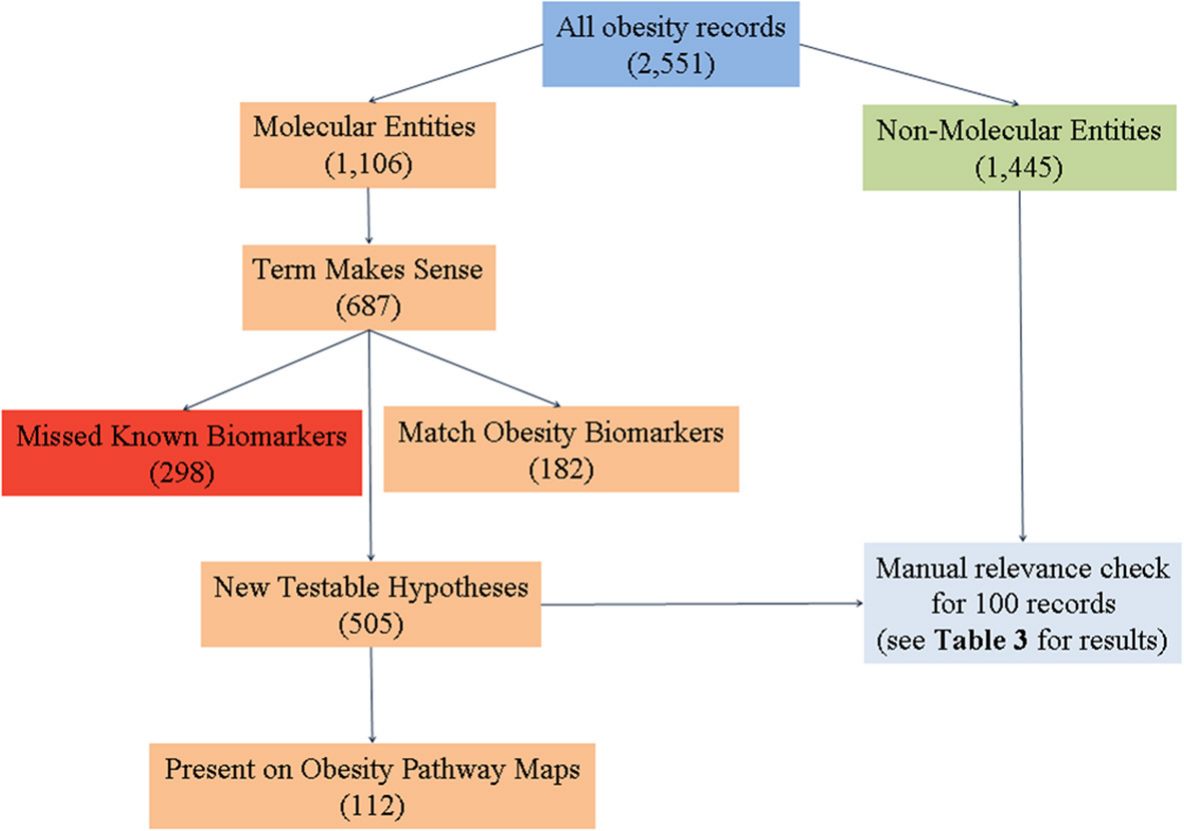
**InfoCodex information map.** InfoCodex information map obtained for the approximately 115,000 documents of the PubMed repository used for the present experiment. The size of the dots in the center of each class indicate the number of documents assigned to it.

As a result of the semantic SOM algorithm, a document collection is grouped into a two-dimensional array of neurons called an information map. Each neuron corresponds to a semantic class; i.e., documents assigned to the same class are semantically similar. The classes are arranged in such a way that the thematically similar classes are nearby (Figure 1).

The described InfoCodex algorithm is able to categorize unstructured information. In a recent benchmark, testing the classification of “noisy” Web pages, InfoCodex reached the high clustering accuracy score F1 = 88% [22]. Moreover, it extracts relevant facts not only from single documents at hand, but it takes document collections as a whole to put dispersed and seemingly unrelated facts and relationships into the bigger picture.

### Text mining biomarkers/phenotypes with InfoCodex

We used the InfoCodex semantic technology for the experiment of finding new biomarkers/phenotypes for D&O by text mining large numbers of biomedical research documents. Five steps were involved:

1. Select a document base and submit it to the InfoCodex semantic engine for text analysis and semantic categorization.

2. Create reference models: teaching the software the essential meaning of “what is a biomarker or a phenotype for D&O.”

3. Determine the meaning of unknown terms (not part of the current ILD) in the document collection by semantic inference using the categorized terms of the ILD.

4. Identify candidates for D&O biomarkers/phenotypes by comparing the subset of documents containing the candidates with the reference models established in Step 2.

5. Compute confidence levels for the identified candidates.

### Step 1: document base

The document base consisted of the following:

• PubMed [9] abstracts with titles: the 115,273 most recent documents (since 1/1/1998) retrieved by the query *diabetes OR obesity OR X* where *X* is a set of 27 known or suspected D&O biomarkers known to Merck and connected by Boolean OR’s (i.e., *X* stands for *5HT2c OR AMPK OR DGAT1 OR FABP_4_aP2 OR FTO OR …*). The 27 biomarkers were supplied by the Diabetes and Obesity Merck franchise and consisted of, predominantly, genes relevant to those disorders.

• Clinical Trials [10] summaries: the 8,960 most recent summaries (since 1/1/2007) retrieved by the query *diabetes OR obesity*. (Adding the 27 Merck D&O biomarkers to the query did not result in any additional hits.)

• Internal Merck research documents, about one page in length: 500 documents. Merck internal research documents refer to a database of full summaries, figures, tables, conclusions, and other key molecular profiling project information predominantly in the fields of atherosclerosis, cardiovascular, bone, respiratory, immunology, endocrinology, diabetes, obesity, and oncology.

### Step 2: reference models

In order to solve the task of the experiment, the InfoCodex semantic engine had to “comprehend” the meaning of biomarker/phenotype for D&O. To this end, a training set of known biomarkers and phenotypes for D&O was determined by naïve (not D&O subject matter experts [SME]) human information research in the literature, independent of the 27 used for the PubMed query. This resulted in a list of 224 reference D&O biomarkers/phenotypes (e.g., “adiponectin” is a biomarker for diabetes, “body mass index” is a phenotype of obesity).

Four subsets of documents were then identified containing these reference terms and “diabetes” or “obesity” (2×2 with biomarkers or phenotypes). Each of these subsets was then clustered into 5–6 subgroups such that the documents in each subgroup were semantically similar to each other using agglomerative hierarchical clustering [23]. As semantic feature vectors (descriptive variables) for the clustering algorithm, the following characteristic document data are used: the probabilities *pt(m)* that a document is categorized by InfoCodex into main topic *m* (*m* = 1 to 15 for the PubMed collection, see Figure 1 for the 15 topics); and the scores for the 15 most important concepts (such as syndromes, biotechnology) resulting from the automatic InfoCodex text analysis for each document. This gives a vector size of 30 components; i.e., two times the number of thematic topics of the information map. The number of 5–6 subgroups was chosen according to the rule of thumb in statistics that the number of subgroups should not exceed *√n* for *n* objects to be clustered. Since *n* ≈ 50 for each of the four subsets, this gives an optimal number of subgroups around 5–6.

For each of the 5–6 sub-clusters, a reference feature vector was then determined for later comparison. This reference feature vector represents essentially an average of the feature vectors of the documents in the sub-cluster, the features being projections onto nodes in the ILD [22]. Each reference feature vector thus encodes one of 5–6 possible meanings of, say, “biomarker for diabetes.”

### Step 3: determination of the meaning of unknown terms

While the ILD contains about 20,000 genes and proteins, it is not guaranteed to identify all the relevant candidates by a simple database look-up. A procedure to infer the meaning of unknown terms from this “hard-wired” knowledge and for synonym analysis [24] had to be devised.

To describe the meaning of an unknown term, a hypernym (superordinate term) is constructed, which corresponds to a known taxon (node) in the taxonomy tree of the ILD. For example, the term “endocannabinoid” is not part of the current ILD and, therefore, its meaning is unknown; but if a procedure can assign the known taxon “receptor” as its most likely hypernym, the unknown term receives a meaning in the sense “is a”.

The taxonomic hypernym is constructed as follows: for each of the unknown terms occurring at least three times in the whole collection, a cross-tabulation is made against all other terms that occur in at least one of the documents containing the unknown term and that are part of the ILD linked to a hypernym. (Example: “unknownword1” occurs in documents 10, 15, and 30. Then, the cross-tabulation is made against all terms occurring either in document 10, 15, or 30). Thereafter, the hypernyms of the most relevant cross-terms are aggregated with the following weighting factors:

• number of occurrences of the cross-terms

• significance of the cross-terms taken from the ILD (each term in the ILD is assigned a significance between 0 and 4)

• *1/entropy* of the cross-terms (terms dispersed over many documents in the collection have a high entropy and thus a low discriminating power)

• correction factor for disjunct neurons, i.e. reduction of the neurons containing either the unknown term or the cross-term by the percentage of the neurons that do not contain both.

Finally, the score of a hypernym is enlarged by partial contributions from the neighboring hypernyms in the taxonomy tree of the ILD (neighbors within the same taxonomy branch). The top scoring hypernym of the cross-terms is selected as the “constructed hypernym” for the unknown term if there is a relatively clear dominance over the other cross-term hypernyms (Table 1).

**Table 1:** InfoCodex computed meanings

***Unknown term***	***Constructed hypernym***	***Associated descriptor 1***
Nn1250	clinical study	insuline glargine
Tolterodine	cavity	overactive bladder
Ranibizumab	drug	macular edema
Nn5401	clinical study	insulin aspart
Duloxetine	antidepressant	personal physician
Endocannabinoid	receptor	Enzyme
Becaplermin	pathology	Ulcer
Candesartan	cardiovascular disease	high blood pressure
Srt2104	medicine	Placebo
Olmesartan	cardiovascular medicine	Amlodipine
Hctz	diuretic drug	Hydrochlorothiazide
Eslicarbazepine	anti nervous	Zebinix
Zonisamide	anti nervous	Topiramate Capsules
Mk0431	antidiabetic	Sitagliptin
Ziprasidone	tranquilizer	major tranquilizer
Psicofarmcolagia	motivation	Incentive
Medoxomil	cardiovascular medicine	Amlodipine

InfoCodex computed meanings of some unknown terms from the experimental PubMed collection.

If no taxonomic hypernym reaches a clear dominance, the descriptors (the most relevant keywords of a document, automatically determined by InfoCodex using the ILD) of the documents containing the unknown term are scored and used to estimate the most likely meaning of the unknown term. The most important descriptor is listed as “associated descriptor 1” in Table 1. It is only used as a substitute in the cases where the described computation of the “constructed hypernym” fails. Although descriptors encode a loose “is related to” association rather than a “is a” hypernym relation, they still provide a useful determination of the meaning of unknown terms when hypernyms cannot be constructed.

The meaning of unknown terms is estimated fully automatically; i.e., no human interventions were necessary and no context-specific vocabularies had to be provided as in most related approaches [6]. The meaning had to be inferred by the semantic engine only based on machine intelligence and its internal generic knowledge base, and this automatism is one of the main innovations of the presented approach. Some of the estimated hypernyms are completely correct: “Hctz” is a diuretic drug and is associated to “hydrochlorothiazide” (actually a synonym). “Duloxetine” is indeed an antidepressant, and the associated descriptor “personal physician” expresses the fact that the contact with the physician plays an important role in (“is related to”) antidepressant usage. Clearly, not all inferred semantic relations are of the same quality.

### Step 4: generating a list of potential biomarkers and phenotypes

Most of the reference biomarkers and phenotypes found in the literature (see Step 2) are linked to one of the following nodes of the ILD:

#### Biomarkers

• *Genes* (including the subnodes “nucleic acids” and “regulatory genes”)

• *Proteins* (including the subnodes “enzymes”, “transferase”, “hydrolase”, ”antibodies”, “simple proteins”)

• *Causal agents* (including subnodes such as “anesthetics”, “diuretic drugs”, “digestive agents”)

• *Hormones*

• *Phenotypes*

• *Metabolic disorders*

• *Diabetes*

• *Obesity*

• *Symptoms* (including the subnode “syndromes”)

Each of the terms appearing in the experimental document base that point to one of these taxonomy nodes, whether via hypernyms given in the ILD for known terms or via constructed hypernyms for unknown terms, are considered as potential biomarker/phenotype candidates. They are assessed by the analysis of the document subsets retrieved from the experimental document base containing a synonym of the candidate in combination with synonyms of “diabetes” or “obesity” respectively. The assembled document subsets are then compared with the previously derived reference models for biomarkers/phenotypes by constructing the corresponding 30-dimensional feature vectors and computing the distances of the descriptive features used for the agglomerative hierarchical clustering. A term qualifies as a candidate for a D&O biomarker or phenotype if most of the semantic similarity deviations from one of the corresponding reference clusters are below a defined threshold (depending on the confidence level described under Step 5).

### Step 5: confidence levels

Not all the biomarker/phenotype candidates established this way have the same probability of being relevant. Therefore, we devised an empirical score representing the confidence level of each term. This confidence measure is based on:

• An initial score derived from the mean deviation of the feature vectors (of the documents retrieved by the *term + synonyms* search) from the closest reference sub-cluster; the smaller the deviation, the higher the confidence

• Up-weighting the confidence score when a large number of documents containing the biomarker/phenotype term/synonyms together with “diabetes” or “obesity” occur in the whole collection.

### Precision/recall against reference vocabularies/databases

The InfoCodex-computed D&O biomarker and phenotype candidates were then compared with Merck internal and external benchmark vocabularies/databases including UMLS [11], GenBank [12], Gene Ontology [13], OMIM [14], and Thomson Reuters [15] D&O biomarker databases according to the following metrics.

• *Precision*: % of InfoCodex outputs matched (defined below) by benchmark biomarkers and phenotypes.

• *Recall*: % of benchmark biomarkers and phenotypes matched by InfoCodex outputs.

• *Novelty*: 100% - precision (i.e., % of InfoCodex outputs not matched by benchmark biomarkers and phenotypes)

These metrics have been used since they are standard measures in pattern recognition and information retrieval. It must be pointed out that in the case at hand they only have a qualitative character as an indicator of emerging trends rather than a precise meaning. On one side, *recall* would only be an accurate measure for the retrieval power if the reference vocabularies were established on exactly the same document corpus used in the experiment. This is not the case, since a comprehensive biomarker repository such as Thomson Reuters’ is based on a broader basis than the 120,000 PubMed abstracts used as a document sample in the current experiment. On the other side, the *novelty* component of a biomarker database is zero (by definition), which makes precision measurements less relevant: Comparing the InfoCodex results with a database of perfect biomarkers the novel candidates will be treated as errors, thereby falsely reducing the precision. This means that the human assessment of valuable and irrelevant novel candidates is the most important result.

Being aware of the limitations of the precision/recall metrics in the case at hand, these standard measures give at least some qualitative indications in the evaluation of the results. The objective of the experiment was not a statistically significant certification of a specific biomarker, but it was a proof-of-concept for the automatic discovery of novel biomarkers/phenotypes. For the purpose of evaluating the efficacy of the proposed semantic methods, the standard precision/recall metrics are nevertheless a useful qualitative measure.

Four different precision and recall scores were computed for all analyses except Thomson Reuters’ (described below), corresponding to a 2x2 of two match types (*exact* and *all = exact + partial*) and two match counting methods (*preferred* and *all = preferred + synonyms*). An example of an *exact* match (ignoring case, spaces, and punctuation) is “diabetes” and “Diabetes”; while “diabetes” and “Diabetes Type 2” is a *partial* match. *Exact* matches are easily computed and do not require curation. Match counting refers to whether synonyms (e.g., “DM2” and “Diabetes Type 2”) and their matches are counted as separate terms (*all = preferred + synonyms*) or conflated with their preferred terms (*preferred*). The most conservative (lowest) estimates of precision and recall are generally *exact/all = preferred + synonyms* and the most liberal (highest) *all = exact + partial/preferred*. This pattern was observed to be fairly robust in our results, so we will report them as this range.

### How reference biomarkers/phenotypes were extracted

#### Merck internal vocabularies

The following dictionaries are not an exhaustive list of Merck internal vocabularies, rather the few we were able to access that contained reference data relevant to the experimental goals.

#### I2E

As stressed above, a really meaningful recall assessment requires a reference list based on the exact same document pool used for the experiment. This is clearly not the case for the available standard databases described below. In order to obtain a rough estimate of such a reference list we used the Merck implementation of Linguamatics I2E [25], a text mining tool, to extract relevant *class1-relation-class2* triples found within sentences in the experimental PubMed collection. This NLP tool provided a more controlled, query-specific method to convert unstructured sentences mentioning biomarkers/phenotypes into a structured term list. It also serves as an example of the typical use of NLP tools as an aid in information extraction of known, lexicalized named entities, for comparison with the associative discovery approach of InfoCodex.

#### I2E-raw

I2E was used to extract relevant *class1-relation-class2* triples found within sentences in the experimental PubMed collection. For biomarkers, *class2* was defined as “diabetes” or “obesity” (note that no synonyms or hyponyms were used) and the *relation* as “biomarker” or any of its synonymous, lexical, or hyponymic variants according to the Linguamatics ontology. *Class1* thus encompassed the I2E-extracted biomarkers. The result was 1,339 such triples; these triples could be de-duplicated, frequency-weighted, and reduced to 788 unique biomarkers for diabetes and 242 for obesity. For example, the sentence “Participants in this sample had insulin resistance, a potent predictor of diabetes” yielded *class1 =* “insulin resistance”; *relation* = “predictive”; *class2* = “Diabetes”.

For phenotypes, *class1* was defined as one of the 27 proprietary Merck-known biomarkers, and the *relation* as “phenotype” or any of its synonymous, lexical, or hyponymic variants according to the Linguamatics ontology. *Class2* thus encompassed the I2E-extracted phenotypes. The result was 18,250 such triples; these could be de-duplicated, frequency-weighted, and reduced to 6,691 unique phenotypes for diabetes and obesity together. For example, the sentence “Constitutively-active AMPK also inhibited palmitate-induced apoptosis” yielded *class1 =* “AMPK”; *relation =* “inhibit”; *class2 =* “apoptosis”.

#### I2E-normalized

The raw I2E phenotype output was normalized by one of Merck’s Linguamatics consultants using automated mapping of the *class2* values to UMLS controlled vocabulary terms, resulting in 12,015 unique triples, or 1,520 unique phenotypes for diabetes and obesity together.

#### I2E-manual

We manually extracted a curated version from the I2E-extracted PubMed sentences. This yielded 3,800 biomarker triples; after de-duplication and synonym/variant conflation, 823 unique biomarkers for diabetes and 315 for obesity. It also yielded 11,365 phenotype triples; after de-duplication and synonym/variant conflation, 4,780 unique phenotypes for diabetes and obesity together.

#### TGI

Merck maintains a Target-Gene Information (TGI) system which includes a database of text-mined and SME-curated binary associations between genes and other biological entities (e.g., between “DGAT1” and “Adipoq”; “Insulin Resistance”; “fatty acid”; “Body mass”; …). From this database we extracted 13,863 binary associations (de-duplicated for case and directionality) in which at least one of the concepts contained at least one of the following strings:

• “diabetes” or “diabetic” (2,014)

• “obese” or “obesity” (2,486)

• one of the 27 Merck D&O biomarkers or their GenBank hyponyms or synonyms (e.g., “AMPK” includes “PRKAA1”; “PRKAA2”; “PRKAB1”; “PRKAB2”; “PRKAG2”; …) (9,363)

### UMLS

We created a version of the UMLS Metathesaurus MRREL (relationship) file (2009AA release) with the terms mapped to the numerical concept identifiers, and from it extracted 205 relationships encoded by different UMLS source vocabularies for the 27 Merck D&O biomarkers and their GenBank synonyms/hyponyms (Table 2).

**Table 2:** UMLS benchmark sources, numbers, and examples

***Source***	***#rels***	***CUI-1***	***concept1***	***rel***	***relationship***	***CUI-2***	***concept2***
NCI	58	C0007595	FABP4 gene	RO	gene_plays_ role_in_process	C1333527	Cell Growth
MSH	45	C0022621	FTO protein, mouse	RN	mapped_to	C2002654	Oxo-Acid-Lyases
OMIM	44	C0064317	KHK gene	RO	related_to	C1416630	Ketohexo-kinase
MTH	38	C0061352	GCGR gene	RO		C1415011	Glucagon Receptor
LNC	20	C0005767	MC4R gene mutation analysis:…	RO	has_system	C1715956	Blood

Sources, numbers, and examples (***concept1***) of benchmark D&O biomarkers/phenotypes extracted from UMLS (CUI: Concept Unique Identifier, RO: Related Other, RN: Related Narrow).

### Gene ontology

We extracted the Gene Ontology (GO) primary relations of the 27 Merck D&O biomarkers and their GenBank synonyms/hyponyms using the GO Online SQL Environment [26]. A primary GO relation involves the GO annotations of the gene itself; for example, {“PRKAA1”, *molecular_function*, “ATP binding”} or {“PRKAA1”, *biological_process*, “fatty acid oxidation”}. Secondary relations were then computed by matching the primary GO terms to a downloaded version of GO. For example, since “PRKAA1” is annotated with “fatty acid oxidation” it would pick up a secondary relation to “fatty acid metabolic process” by virtue of the internal GO relation {“fatty acid oxidation”, *is_a*, “fatty acid metabolic process”}. The result was 4,104 primary and 3,688 secondary GO reference D&O biomarkers/phenotypes.

### OMIM

Disease-gene links in the Online Mendelian Inheritance in Man (OMIM) database were manually extracted for the 27 Merck D&O biomarkers and their GenBank synonyms/hyponyms, yielding 41 reference biomarkers/phenotypes, such as:

• D&O biomarker/hyponym: MC4R

• OMIM gene ID: 155541

• OMIM disease ID: 601665

• Disease name: OBESITY; LEANNESS, INCLUDED

• Disease-gene links: OB4, OB10Q, PPARGC1B, FTO, BMIQ8, GHRL, SDC3, …

### Thomson Reuters

Thomson Reuters SMEs compared the InfoCodex PubMed output to their proprietary biomarkers and signalling pathways for obesity, diabetes mellitus type 1 (DM1), diabetes mellitus type 2 (DM2), and diabetes insipidus (DI) from MetaBase, a systems biology database developed in GeneGo (now Thomson Reuters). Biomarkers for abovementioned disorders were annotated in the scope of the disease consortium MetaMiner Metabolic Diseases, a partnership between Thomson Reuters, pharmaceutical companies and academia focused on development of systems biology content for disease research in the form of disease biomarkers, disease pathway maps, and disease data repositories. A biomarker in MetaMiner programs is defined as any molecular entity (DNA, RNA, protein, or an endogenous compound) that is distinctly different between normal and disease states. A gene can be classified as a biomarker if the evidence is established on at least one of the following levels: DNA (e.g. mutations, rearrangements, deletions), RNA (e.g. altered expression level, abnormal splice variants) or protein (e.g. change in abundance, hyperphosphorylation). Disease specific pathway maps developed in MetaMiner consortia depict signalling events most relevant for a disease in focus as well as showing the changes in normal pathways that occur in disease states (e.g., gain and loss of protein functions resulted in new or disrupted protein interactions). All pathway maps developed in the scope of MetaMiner programs are subjected to approval and review of consortia members who are experts in the corresponding disease areas.

**Figure 2:**
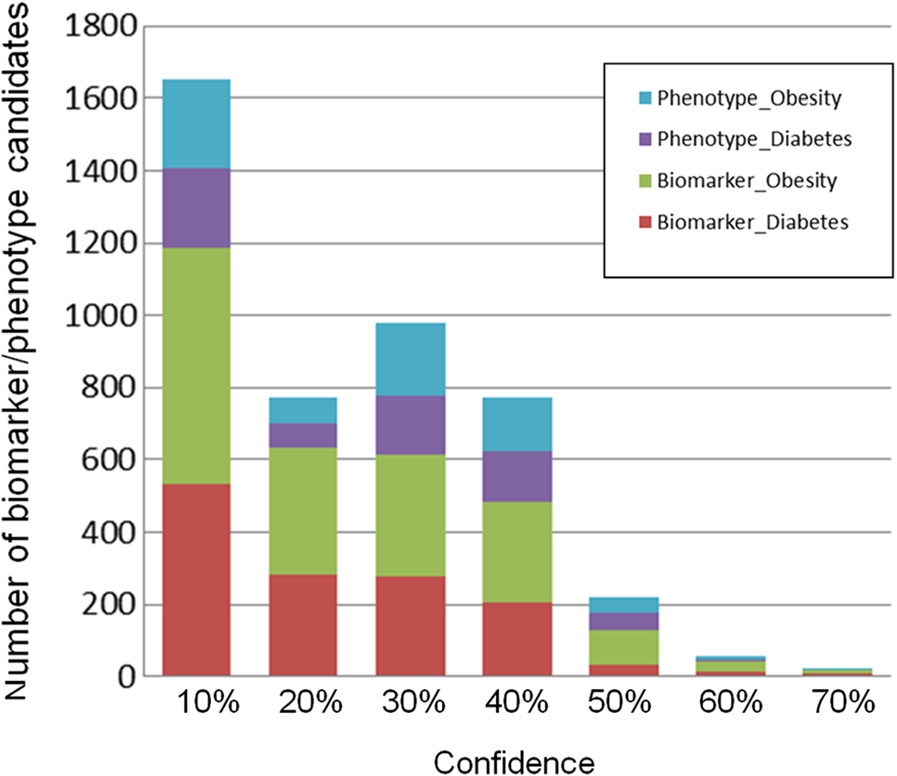
**Thomson Reuters obesity algorithm.** Obesity example of Thomson Reuters algorithm for scoring matches between InfoCodex output (“All obesity records”) and Thomson Reuters knowledge bases.

After performing the comparisons, Thomson Reuters reported matching statistics according to the algorithm shown in Figure 2. In Figure 2 it can be seen that precision and recall can be computed for obesity from the “All [InfoCodex] obesity records”; “Match Thomson Reuters Obesity Biomarkers”; and “Missed Known Biomarkers”: precision = 182/2,551 = 7%; recall = 182/(182 + 308) = 37%. (It has to be kept in mind that the computed precision/recall values are just an indication and not an accurate measure as explained above.) “Relevance” and “Sense checking” refer to an effort to narrow the novelty (93%) down to useful novelty: 512 (20%) “New testable hypothesis” of which 71 (3%) appear to be supported by the candidate biomarker’s presence on the Thomson Reuters Obesity Pathway Maps.

### Merck SME qualitative analysis

Of particular interest to Merck was the question “What biomarker/phenotype terms could be identified by the semantic engine that are in the Merck internal research documents and not publicly available in PubMed and ClinicalTrials.gov?” Creating this “unique to Merck” list was an exercise in cross referencing the three engine-produced lists for PubMed, ClinicalTrials.gov, and Merck internal research documents to uncover the terms in one list (Merck internal research documents) that are not in the other two lists (PubMed and ClinicalTrials.gov). The complete “unique to Merck” list was then culled of terms that were clearly not biomarkers/phenotypes and/or too general to be considered valuable medical terms.

## Results

### Overall output

**Figure 3:**
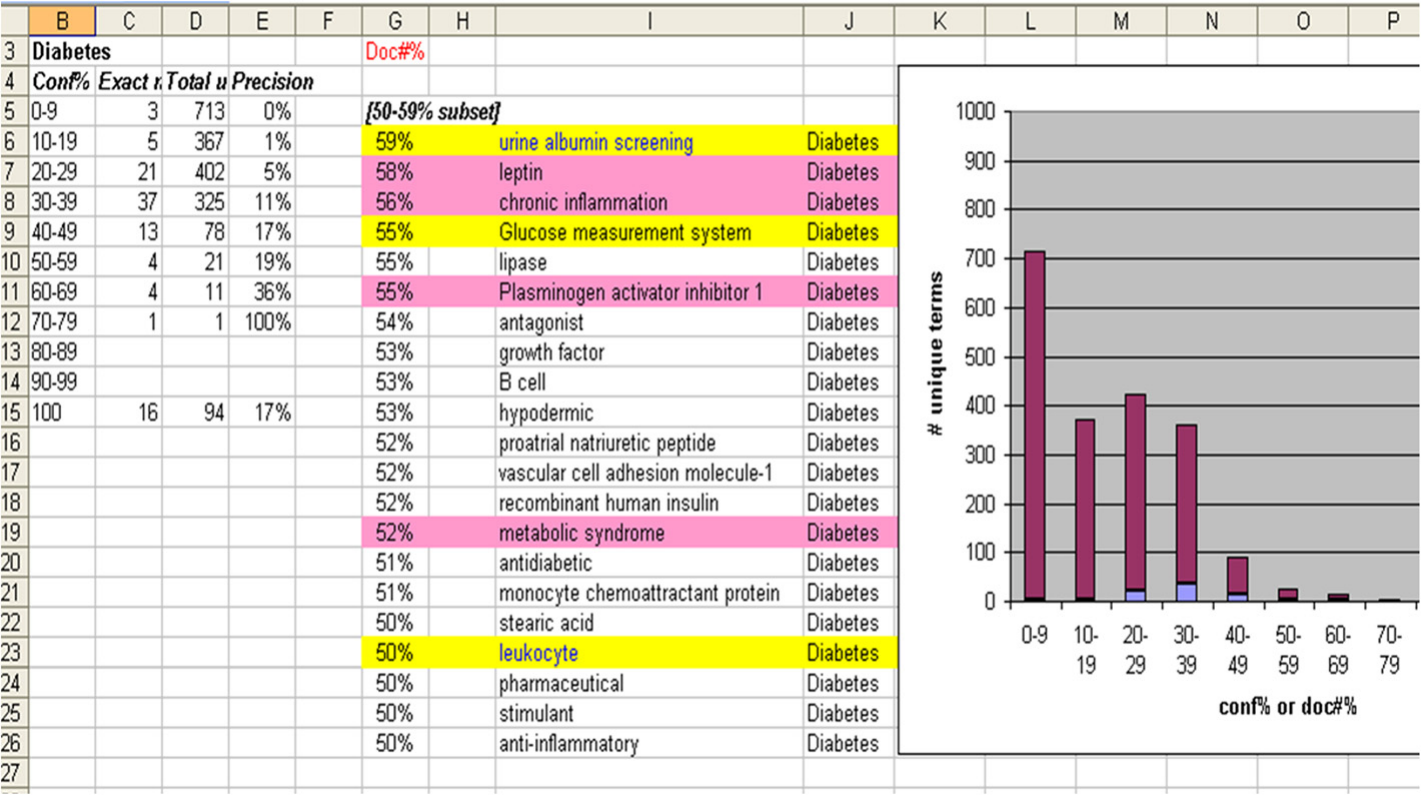
**PubMed results confidence level distribution.** Confidence level distribution of candidates discovered by InfoCodex text mining of the experimental PubMed collection.

The InfoCodex output was transformed into lists of D&O biomarker/phenotype candidates with their confidence level (CL) scores and other metadata. A total of 4,467 {*entity, biomarker/phenotype, diabetes/obesity*} candidate triples were found (1,361 and 1,743 biomarkers for diabetes and obesity, respectively, and 653 and 710 phenotypes for diabetes and obesity, respectively) ranging in CL from 3% to 70%, and distributed as shown in Figure 3. The highest scoring candidates discovered by InfoCodex text mining of the experimental PubMed collection are shown in Table 3.

**Table 3:** PubMed results with highest confidence levels

***Row***	***Term (A)***	***Relationship (B)***	***Object (C)***	***Conf% (D)***	***#Docs (E)***	***PMIDs (F)***
1	glycemic control	BiomarkerFor	Diabetes	70.3	1122	20110333, 20128112, 20149122,
2	Insulin	PhenoTypeOf	Diabetes	68.3	5000	19995096, 20017431, 20043582,
3	Proinsulin	BiomarkerFor	Diabetes	67.8	105	16108846, 9405904, 20139232,
4	TNF alpha inhibitor	PhenoTypeOf	Diabetes	67.1	245	9506740, 20025835, 20059414,
5	anhydroglucitol	BiomarkerFor	Diabetes	67.1	10	20424541, 20709052, 21357907,
6	linoleic acid	BiomarkerFor	Diabetes	67.1	61	20861175, 20846914, 15284064,
7	palmitic acid	BiomarkerFor	Diabetes	67.1	24	20861175, 20846914, 21437903,
8	pentosidine	BiomarkerFor	Diabetes	67.1	13	21447665, 21146883, 17898696,
9	uric acid	BiomarkerFor	Obesity	66.8	433	10726195, 19428063, 10904462,
10	proatrial natriuretic peptide	BiomarkerFor	Obesity	66.6	4	14769680, 18931036, 17351376,
11	ALT values	BiomarkerFor	Diabetes	66.3	2	20880180, 19010326
12	adrenomedullin	BiomarkerFor	Diabetes	64.3	7	21075100, 21408188, 20124980,
13	fructosamin	BiomarkerFor	Diabetes	64.2	59	20424541, 21054539, 18688079,
14	TNF alpha inhibitor	BiomarkerFor	Diabetes	62.1	245	9506740, 20025835, 20059414,
15	uric acid	BiomarkerFor	Diabetes	61.8	259	21431449, 20002472, 20413437,
16	monoclonal antibody	BiomarkerFor	Obesity	61.7	41	14715842, 21136440, 21042773,
17	Insulin level QTL	PhenoTypeOf	Obesity	61.2	1167	16614055, 19393079, 11093286,
18	stimulant	BiomarkerFor	Obesity	61.2	646	18407040, 18772043, 10082070,
19	IL-10	BiomarkerFor	Obesity	60.9	120	19798061, 19696761, 20190550,
20	central obesity	PhenoTypeOf	Diabetes	59.5	530	16099342, 17141913, 15942464,
21	lipid	BiomarkerFor	Obesity	59.5	4279	11596664, 12059988, 12379160,
22	urine albumin screening	BiomarkerFor	Diabetes	59.0	95	20886205, 19285607, 20299482,
23	tyrosine kinase inhibitor	BiomarkerFor	Obesity	58.8	83	18814184, 9538268, 15235125,
24	TNF alpha inhibitor	BiomarkerFor	Obesity	58.0	785	20143002, 20173393, 10227565,
25	fas	BiomarkerFor	Obesity	57.7	179	12716789, 17925465, 19301503,
26	leptin	PhenoTypeOf	Diabetes	57.6	870	11987032, 17372717, 18414479,
27	ALT values	BiomarkerFor	Obesity	57.4	8	16408483, 19010326, 17255837,
28	lipase	BiomarkerFor	Obesity	56.8	356	16752181, 17609260, 20512427,
29	insulin resistance	PhenoTypeOf	Obesity	55.8	5000	20452774, 20816595, 21114489,
30	chronic inflammation	PhenoTypeOf	Diabetes	55.7	154	15643475, 18673007, 18801863,

Highest confidence level scoring biomarker/phenotype candidates discovered by InfoCodex text mining of the experimental PubMed collection. The identified candidate terms appear in column A, with their relationship to diabetes or obesity in columns B-C. The confidence level, in column D (the descending sort key), is normalized on a scale in which the maximum of 100% is the score of the manually curated reference biomarkers/phenotypes. In column E are the numbers of documents in which a given candidate term appears. Column F displays the PubMed IDs of the most relevant PubMed documents for purposes of manual SME review. Note that the same term can have multiple entries since it can have different relationships (biomarker for diabetes, phenotype for obesity, etc.).

### Precision/recall

The fine conceptual/definitional difference between “biomarkers” and “phenotypes” was evident in the high degree of overlap in the two subsets produced by InfoCodex and I2E. Therefore we combined them for purposes of computing precision and recall. The results are shown in Table 4. Due to the volume of data and the need for SME curation of partial matches, we could not compute values for all of the quadrants of the 2×2 matching matrix described under Methods. The numbers tend to be low but there were some encouraging trends. InfoCodex precision/recall was higher for the more reliable manually parsed I2E output than for raw or auto-normalized I2E output, and could be made even higher by principled lumping of I2E terms (e.g., lumping *hyperglycemia, postprandial hyperglycemia, chronic hyperglycemia, hyperglycemia in women,* etc.). The high-end of the recall score ranges had good consistency for the most reliable benchmarks (I2E manual 33%, UMLS + GO + OMIM 35%, Thomson Reuters 36%).

**Table 4:** Precision and recall

***Benchmark***	***Benchmark corpus***	***InfoCodex corpus***	***Precision***	***Recall***
I2E raw	PubMed	PubMed	(exact)	(exact)
			<1% obesity	5% obesity
			3-5% diabetes	9-11% diabetes
			3-7% MDOB	7% MDOB
I2E normalized	PubMed	PubMed	(exact)	(exact)
			3-7% MDOB	3-7% MDOB
I2E manual	PubMed	PubMed	1-5% obesity	9-33% obesity
			3-11% diabetes	9-31% diabetes
			3-26% MDOB	4-15% MDOB
UMLS + GO + OMIM	UMLS + GO + OMIM	PubMed	1-4%	3-22%
			1-8% (unary)	4-35% (unary)
Thomson Reuters	Thomson Reuters	PubMed	7-36% obesity	36% obesity
				18% DM2
			9-49% DM2	22% DM1
				25% DI
TGI	TGI	PubMed	0-5% obesity	(exact) 2.5%
			0-4% diabetes	
			1-14% MDOB	
I2E manual	PubMed	ClinicalTrials.gov	(preferred terms) 27-59%	(preferred terms) 3-7%
UMLS + GO + OMIM	UMLS + GO + OMIM	ClinicalTrials.gov	(preferred terms) 1-2%	(preferred terms) <1%
I2E manual	PubMed	Merck internal	(preferred terms) 8-14%	(preferred terms) 1-2%
UMLS + GO + OMIM	UMLS + GO + OMIM	Merck internal	(preferred terms) <1%	(preferred terms) <1%

Precision and recall of InfoCodex candidate biomarkers/phenotypes compared to various benchmarks. “(exact)” and “(preferred terms)” refer to sub-ranges according the 2x2 matching matrix described in the text under “Methods – Precision/recall”. “MDOB” refers to the InfoCodex output subset containing references to the 27 Merck D&O biomarkers. “(unary)” means all InfoCodex candidate biomarkers/phenotypes were lumped together across obesity, diabetes, and MDOB, in contrast to the default binary criterion for matching.

**Figure 4:**
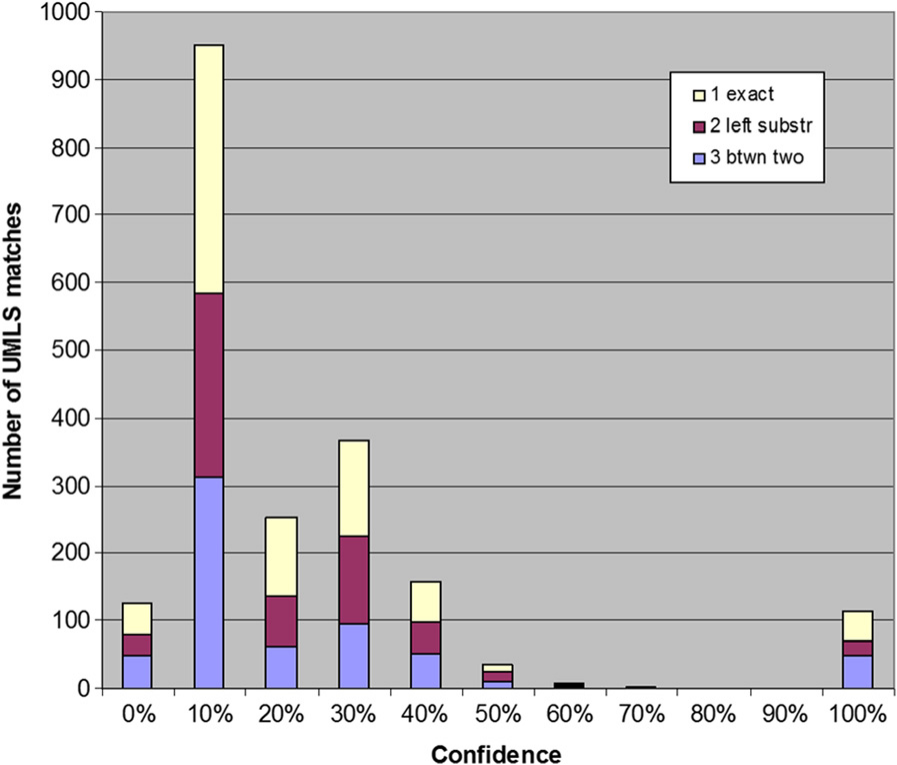
**PubMed results confidence levels x I2E-manual precision.** Correlation between InfoCodex confidence levels (Conf%; purple bars) and precision (light blue bars) against I2E-manual diabetes PubMed benchmark. Pink shading: exact match; yellow shading: partial match. Row 15 (100 Conf%) represents a member of the manually compiled reference set.

The precision scores for individual biomarkers were highly variable, but some were impressive (I2E manual 52%, Thomson Reuters 49%, TGI 35%, ClinicalTrials.gov 59%) (not shown). For diabetes, there was a slight correlation between InfoCodex confidence level (CL) scores and precision against the I2E-manual benchmark (Figure 4). However, among the novel subset, there appeared to be a slight *inverse* correlation between quality and CL (see next section).

### Novelty quality

Novelty is the “flip side” of precision; the “bad news” of low precision is accompanied by the “good news” of high novelty. But novel biomarker/phenotype candidates are useful only if they are high quality (credible enough to justify follow-up research). Row 18 (“stimulant”) in Table 3 and “antagonist” and “hypodermic” in Figure 4 would appear to be examples of low quality candidates. On the contrary, “insulin” (Row 2 in Table 3) and “proinsulin” (Row 3 in Table 3) are positive examples of proper candidates recognized as known biological complexes of diabetes. According to the classification of type 1 and type 2 diabetes adopted by the World Health Organization – a loss of the physical or functional β-cell mass and increased need for insulin due to insulin resistance, respectively – it is quite possible that both processes would operate in a single patient and contribute to the phenotype of the patient [27]. Fasting intact proinsulin is a reliable and robust biomarker for beta-cell dysfunction, metabolic insulin resistance, and cardiovascular risk in Type 2 diabetes mellitus patients [28].

### Associative retrieval of known D&O biomarkers/phenotypes

In an effort to exemplify the associative recovery of a known phenotype of obesity, we used PubMed as a baseline to characterize the retrieval of a term InfoCodex specified as a phenotype. Melatonin receptor 1B (MTNR1B) is a candidate gene for type 2 diabetes acting through elevated fasting plasma glucose (FPG). As a phenotype of obesity, MTNR1B should not be considered novel, but it can be used to substantiate the soundness of InfoCodex results extracted from PubMed and to illustrate the associative retrieval mechanism.

In PubMed, a search for “MTNR1B” AND “obesity” returned 9 documents, of which two (PMID: 20200315, 19088850) matched the PubMed abstracts selected by InfoCodex to substantiate its identification of MTNR1B as an obesity phenotype. When the criterion “phenotype” was added to the search, however, PubMed did not return any documents. A simple PubMed search would have thus failed to immediately identify MTNR1B as an obesity phenotype.

In PMID 19088850, the word “phenotyping” is used to describe an action on a cohort of subjects, not a specification of MTNR1B as a phenotype. Later in the abstract the word “traits” is, however strongly indicating MTNR1B as a phenotype of obesity. The word “phenotype” is missing entirely in PMID 20200315. The InfoCodex semantic engine could still correctly combine the MTNR1B-related information “increased prevalence of obesity” in PMID 20200315 with “traits” in PMID 19088850 to infer MTNR1B as a phenotype of obesity. A human read of these two abstracts would indeed immediately detect MTNR1B as a phenotype for obesity, an identification the PubMed search engine failed to reveal, while the InfoCodex semantic engine was able to reconstruct it by integrating information distributed over the two documents even if the exact word “phenotype” never appears in relation to MTNR1B. Two abstracts subsequently indexed by PubMed also fully confirm the identification of MTNR1B as a phenotype for obesity.

In this MTNR1B benchmark set, the comparison with another, traditional text mining approach (i.e., PubMed Search) exposed a relevant difference in results. The measured InfoCodex CL for MTNR1B as a phenotype of obesity is 3.6%. This low CL for a term is consistent with the high plausibility/interestingness observation addressed in the Discussion section.

### Thomson Reuters relevance analysis

Thomson Reuters D&O SMEs quantified novelty quality as shown by the “Relevance” and “Sense checking” components of Figure 2. In that case, Thomson Reuters analysts narrowed 2,369 (93%) novel obesity biomarker candidates down to 512 (20%) credible molecular biomarkers, of which 71 (3%) appeared to be initially confirmed by their presence on the Thomson Reuters Obesity Pathway Maps. For the finer relevance analysis, random samples of high- and low-confidence level InfoCodex/PubMed biomarker candidates were scored on the relevance scale from 0 to 10 as shown below (several thresholds of the scale below 10 reflect main types of erroneous associations between found biomarkers and diseases and how close they are in our opinion to relevant and unambiguous relationships):

• 10 – totally relevant and unambiguous relationship

• 8–9 – relevant, but can be associated with a related term – disease subtypes, disease symptom or consequence, etc.

• 6–7 – relevant, but correlation is rather remote. For example, some drugs may be causing elevation of blood pressure and should be administered with caution in diabetes patients (but drug is not for diabetes)

• 4–5 – associated in a specific context or found only one record

• 1–3 – low level of association

• 0 – no association, or term is so general it is not going to make sense

From the results in Table 5, it can be seen that only the obesity/molecular samples had respectable average relevance scores (6.9 high confidence, 6.2 low confidence). DM2/molecular and obesity/non-molecular terms averaged around 3 for both low and high confidence. DM2/non-molecular and both classes of DM1 exhibited an *inverse* confidence score effect, averaging around 1 for high and 3.4 for low. The main reason for low scores of non-molecular biomarkers with high confidence scores is the high percentage of terms that were considered to be too general and received score of 0; for example, “drug delivery”, “first-in-class”, “genotyping” and others.

**Table 5:** SME relevance analysis

***Biomarker type / disease***	***Average relevance scores for high confidence candidates***	***Average relevance scores for low confidence candidates***
**Molecular Biomarkers**		
Diabetes Type 1	1.6	3.2
Diabetes Type 2	3.6	3.7
Obesity	6.9	6.2
**Non-molecular Biomarkers**		
Diabetes Type 1	0.7	3.4
Diabetes Type 2	0.9	3.6
Obesity	2.6	2.8

Scale is described in main text.

### UMLS mapping

A second approach to assessing the quality of the novel InfoCodex biomarker/phenotype candidates was mapping them to UMLS by co-sorting with the full 2009AA UMLS English lexicon extracted from the MRCONSO file. Three types of matches could be computed from this sort without SME curation: exact, left substring, and “between 2” (all ignoring case, spacing, and punctuation), as exemplified in Table 6. Exact matches are clear evidence of plausibility from a lexicosemantic type point of view (as opposed to the D&O SME point of view of the Thomson Reuters analysts), while the other two match types vary.

**Table 6:** UMLS mapping

***Match type***	***InfoCodex novel biomarker/ phenotype candidate***	***UMLS term***
Exact	ABCC8 gene	ABCC8 gene
Left substring	ABCC8	ABCC8 gene
	9-cis-retinoic acid	9-cis-retinoic acid biosynthesis
	Cara	C ara A
	*CD-1*	*CD100 antigen*
Between 2 (;; = separator)	acute coronary syndromes	Acute Coronary Syndrome ;; Acute coronary thrombosis…
	abnormal laboratory findings	Abnormal Keratinocyte ;; Abnormal Laboratory Result (Biochemistry)
	*alpha receptor*	*Alpha Rays ;; alpha resorcylic acid*
	*Bmisds*	*BM Iron Stain Test ;; BmJHE*

Examples of novel InfoCodex biomarker/phenotype candidates mapped to UMLS by three uncurated match types. The italicized matches are clearly false (unrelated conceptually).

The results are shown in Table 7. The highest percentage of exact matches was found for the novel InfoCodex biomarker/phenotype candidates from ClinicalTrials.gov (52%), followed by PubMed (39%), and lastly by Merck internal research documents. This order “makes sense” because new knowledge generally takes time to become canonical enough for controlled vocabularies. Clinical trials would be expected to be founded on the oldest, most well-developed knowledge, while Merck internal research concerns the newest and most tentative, with published literature being intermediate, consistent with our UMLS exact match results.

**Table 7:** UMLS match type distribution

***Corpus***	***Exact***	***Left substring***	***Between 2***
Pubmed	789 (39%)	591 (29%)	632 (31%)
ClinicalTrials.gov	409 (52%)	225 (29%)	155 (20%)
Merck internal	24 (28%)	25 (29%)	38 (44%)

UMLS match type distribution of novel InfoCodex biomarker/phenotype candidates from the three corpora analyzed.

**Figure 5:**
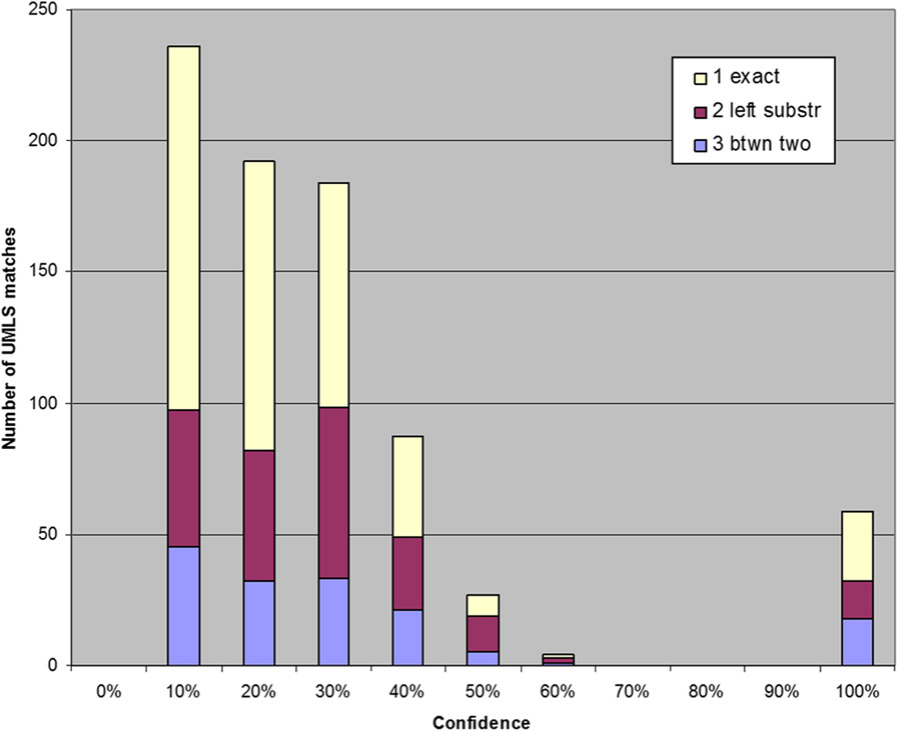
**PubMed results confidence levels x UMLS match type.** Confidence levels of novel InfoCodex biomarker/phenotype candidates from PubMed broken down by match type to UMLS terms (100% refers to the manually discovered reference/training set).

**Figure 6:**
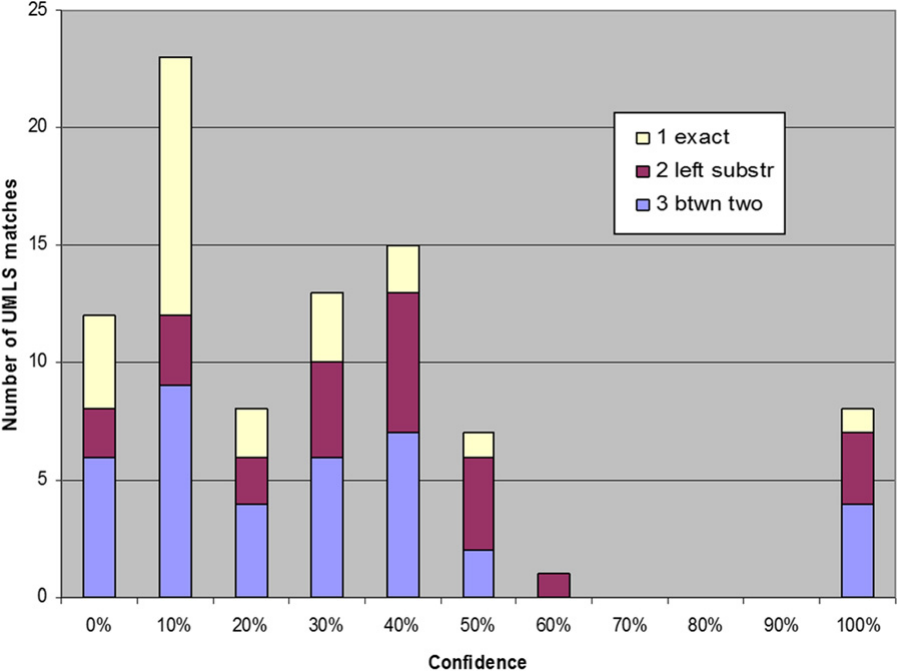
**ClinicalTrials.gov results confidence levels x UMLS match type.** Confidence levels of novel InfoCodex biomarker/phenotype candidates from ClinicalTrials.gov broken down by match type to UMLS terms (100% refers to the reference/training set).

**Figure 7:**
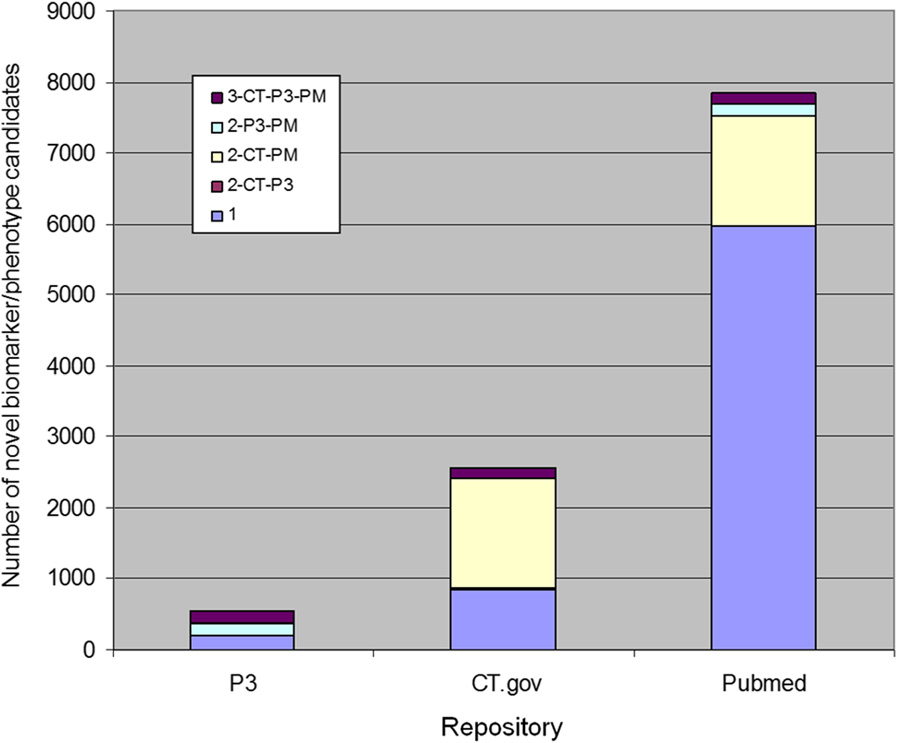
**Merck P3 results confidence levels × UMLS match type.** Confidence levels of novel InfoCodex biomarker/phenotype candidates from Merck internal research documents broken down by match type to UMLS terms (100% indicates the reference/training set).

We further broke down the novel InfoCodex biomarker/phenotype candidates by confidence level and their mapping to UMLS as shown in Figures 5, 6, 7. For the PubMed candidates there was little or no confidence level effect, but there appeared to be an *inverse* correlation (more exact matches at lower confidence levels) for the ClinicalTrials.gov and Merck internal candidates.

### Merck SME qualitative results

**Figure 8:**
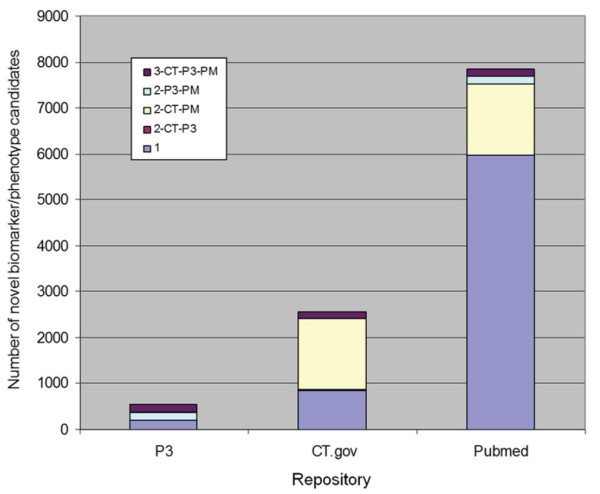
**Novel candidates repository overlap.** Overlap between novel InfoCodex biomarker/phenotype candidates from PubMed (PM), ClinicalTrials.gov (CT), and Merck internal research documents (P3). Lavender shading: found in one repository only; dark violet shading: found in all three; others: found in two.

10,953 novel biomarker/phenotype candidate terms were identified by InfoCodex from PubMed, ClinicalTrials.gov, and Merck internal research documents (“P3” in the figures). The summary for each data source and the overlap across data sources is summarized in Figure 8.

Table 8 shows some examples of novel InfoCodex biomarker/phenotype candidates from Merck internal research documents that were clearly not biomarkers/phenotypes and/or too general to be considered valuable medical terms. Confidence levels reach the 50% + range in the example presented. Tens of interesting, plausible biomarkers/ phenotypes were found (not shown due to proprietary nature) in Merck internal research documents database (P3) but not in PubMed or ClinicalTrials.gov. These interesting, plausible terms are expressed with low CLs (<15%) and document counts (<7). This paradoxical phenomenon – inverse relationship between plausibility/interestingness and confidence levels (as well as document counts) – is discussed in the next section.

**Table 8:** UMLS benchmark sources, numbers, and examples

***Term***	***Relationship***	***Object***	***Target***	***Conf%***	***#Docs***
wenqing	BiomarkerFor	Obesity	Obesity	53.5	29
proteomic	BiomarkerFor	Obesity	Obesity	40.8	128
gene expression	BiomarkerFor	Obesity	Obesity	38.9	62
Mouse model	BiomarkerFor	Obesity	Obesity	19.8	17
muise	BiomarkerFor	Obesity	Obesity	17.5	20
athero-	BiomarkerFor	Obesity	Obesity	16.5	6
shrna	BiomarkerFor	Obesity	Obesity	9.6	4
inflammation	BiomarkerFor	Obesity	Obesity	8.2	4
TBD	BiomarkerFor	Obesity	Obesity	7.4	3
body weight	PhenoTypeOf	Diabetes	MGAT2		1
cell line	BiomarkerFor	Diabetes	MGAT2		1

Examples of uninteresting novel InfoCodex biomarker/phenotype candidates from Merck internal research documents.

## Discussion

One of the major high-level novelties of this experiment with respect to other recent studies [6] lies in the fact that the experiment was designed to test the power of autonomous self-organizing semantic engines. By the analysts, the experiment was handled strictly as a “blind experiment” and no feedback from preliminary results was used to improve the machine-generated results.

Compared with recent studies [29-32] aimed at the extraction of drug–gene relations from the pharmacogenomic literature, our experiment introduces three novelties. First, while most related work is based on high-quality manually curated knowledge bases such as PharmGKB [29] to train the recognition of connections between specific drugs and genes, our experiment’s reference/training set (Step 2) was assembled in an *ad hoc* way by naïve (non-biologist) PubMed searchers. Second, aside from the generic ontology in the ILD, no context-specific vocabularies (e.g., UMLS) were provided to inform the semantic engine. The meaning of unrecognized words had to be inferred by the InfoCodex engine based only on its universal internal linguistic database. Third, the text mining algorithms used here do not use rule-based approaches [31], or analyze co-occurrences sentence by sentence [29] or section by section [32], but rather they extract knowledge from entire documents and their relations with semantically related documents.

Natural language processing (NLP) approaches extract possible relations through analyzing documents sentence by sentence. Basically, such techniques can detect only those relations that have been written down by an author in some form or another, i.e., that are already known to some extent. Discoveries of really novel relationships require more than a sentence-by-sentence analysis. They are rather a result of the combination of small, seemingly unrelated and unnoticed facts dispersed over isolated publications. This is exactly what the InfoCodex approach intends to achieve, combining semantic technologies with statistical and neural analysis of whole document collections.

Among the discovered potential biomarkers/phenotypes there are some candidates of apparent high quality (“needles in the haystack”). Some of these have been tested, with encouraging results, for actual novelty in a very preliminary way by internet searches (e.g., “xyz obesity” in Google or PubMed) where “xyz” is one of the candidates and “actual novelty” is defined as low hit rates, near or at zero, compared to known biomarkers (e.g., “adiponectin obesity”) with hit rates in the hundreds of thousands. More rigorous testing will require sizable effort and so we leave it up to future follow-up studies.

However, most results are not plausible or incompletely specified. This is not surprising for the following reasons:

• No prior knowledge on biomarkers/phenotypes was provided to the analysts who assembled the reference/training set (Step 2) and re-iteration was not allowed.

• Domain-specific knowledge (e.g., UMLS) was not added to the ILD to help the clustering or term extraction processes.

• Although it is certainly true that a large amount of human work was required to assess the quality of the generated results for potential novel biomarkers/ phenotypes in the proof-of-concept phase, the semantic analysis process for a discovery of novel biomarkers was largely automatic. No human expert feedback was allowed to influence the results. According to the blind nature of the experiment, the pure machine intelligence has been tested.

In view of these constraints, the capability of automatically identifying high quality candidates is encouraging. The machine discovery process can deliver a list of potential biomarkers and can aid the biomarker discovery process by prioritizing them for follow-up research by confidence scores.

On the basis of the quality assessment by human SMEs, the quality of the machine discovery could substantially be improved by the following measures:

• Utilization of reliable SME-curated training sets of biomarkers/phenotypes for the construction of the reference models (Step 2 above). In the present blind experiment the absence of any prior knowledge has led to a poor choice of some of the reference sets (e.g., generic terms such as “transforming growth factor” or “epistatic interaction” for biomarkers).

• Putting the focus of the novel biomarker discovery on proteins and genes as specified by the ILD ontology and giving other terms a lower weight.

• Extension of the ILD with additional proteins and genes taken from well-recognized biomedical dictionaries (e.g., UMLS), thus reducing the uncertainty in estimating the meaning of unknown terms and avoiding the use of incompletely specified terms.

• Use of named entity extraction rules to enhance the mapping of incomplete terms to complete, standardized biological terms.

• Improvement of the scoring method used in the estimation of the confidence level. The number of documents in which a particular biomarker/phenotype candidate appears should not be used to up-weight the confidence score, since it enhances too much the importance of irrelevant generic terms appearing in many documents (Step 5 above). A relevant quantity to include in the confidence score design is the information-theoretic entropy of candidates or – even more important – the joint entropy between the distribution of the candidates and the reference models over the neurons.

## Conclusions

The reported approach of employing autonomous self-organising semantic engines to aid biomarker discovery shows promise and has potential to impact pharmaceutical research; for example to shorten time-to-market of novel drugs, or for early recognition of dead ends such as those with prohibitive side-effects through targeted extraction of relevant information.

The machine discovery must be considered as a semi-automatic, rather than a fully automatic, process since it cannot fully replace the competence of human researchers. The most promising approach is a hybrid process in which the automatically inferred discoveries are assessed by human experts. Sorting results by a measure of confidence would significantly speed the review process for the highest/lowest ranges in the scale. The verification of the identified candidates can be supported by InfoCodex’s user interface, visualizing the semantic similarity between a query text and the retrieved documents.

In conclusion, we stress that what we presented here is a first step in an iterative process in which the machine discovery of biomarkers/phenotypes and related pharmacogenomic entities is perfected to a level sufficient for human assessment of only the top tier of proposed novel entities. The final machine process we have in mind should not only lead to cost cutting with respect to traditional human research but it could become a valuable ingredient to tackle the sheer number of relevant documents available.

## Abbreviations

ADME: Absorption/Distribution/Metabolism/Excretion; CL: Confidence Level; CRO: Contract Research Organization; CUI: Concept Unique Identifier; D&O: Diabetes & Obesity; DM1: Diabetes Mellitus Type 1; DM2: Diabetes Mellitus Type 2; DI: Diabetes Insipidus; GO: Gene Ontology; ILD: InfoCodex Linguistic Database; IR: Information Retrieval; NLP: Natural Language Processing; NMR: Nuclear Magnetic Resonance; OMIM: Online Mendelian Inheritance in Man; P3: Merck Internal Research Documents Database; RO: Related Other; RN: Related Narrow; SME: Subject Matter Expert; SOM: Self-Organizing Map; TGI: Target-Gene Information.

## Competing interests

The authors’ corporate affiliations are given on the title page. Merck & Co., Inc., provided funding and computing resources for the work reported here.

## Authors’ contributions

CAT supported the development of specialized algorithms described under “Text mining biomarkers/phenotypes with InfoCodex” and wrote the first draft of this report. CW served as InfoCodex representative on the Merck project team. DP was the Merck project leader and driving force, and coordinated the joint writing of this report. MES did the precision/recall evaluations and detailed editing of report drafts. SB served as Thomson Reuters representative on the Merck project team and coordinated the Thomson Reuters SME analyses. All authors read and approved the final manuscript.

## Authors’ information

CAT holds a PhD in Theoretical Physics and a Masters in Economics. His work on Quantum Information Theory has been publicized in the press, including Nature Science Update.

CW holds a PhD and MSc in Physics. He has been involved in the development of semantic technologies for knowledge extraction, clustering and categorization for over ten years.

DP holds a BS in Engineering and an MBA in Finance. He has developed and supported laboratory basic research and pre-clinical applications for MRL for the past 15 years.

MES holds a MA in Biochemistry and a PhD in Information Science. He has been involved in biomedical vocabularies, ontologies, and search at NIH, NLM, and Merck since 1988.

SB holds a PhD in Biochemistry and BS in Economics. She has been involved in curation of scientific information and development of knowledge bases and data mining software in Thomson Reuters since 2006.
